# Alterations of gut microbial pathways and virulence factors in hemodialysis patients

**DOI:** 10.3389/fcimb.2022.904284

**Published:** 2022-08-26

**Authors:** Xiaochun Shi, Bei Gao, Anvesha Srivastava, Zahra Izzi, Yoosif Abdalla, Weishou Shen, Dominic Raj

**Affiliations:** ^1^ Department of Environmental Ecological Engineering, School of Environmental Science and Engineering, Nanjing University of Information Science and Technology, Nanjing, China; ^2^ School of Marine Sciences, Nanjing University of Information Science and Technology, Nanjing, China; ^3^ Division of Kidney Diseases and Hypertension, George Washington University School of Medicine, Washington, DC, United States; ^4^ Langley High School, McLean, VA, United States; ^5^ Jiangsu Key Laboratory of Atmospheric Environment Monitoring and Pollution Control, Collaborative In-novation Center of Atmospheric Environment and Equipment Technology, Nanjing, China

**Keywords:** random forest, hemodialysis, virulence factor, metagenome, microbiome

## Abstract

Alterations in gut microbiota might contribute to uremic toxicity and immune dysregulation in patients with end-stage renal disease. Hemodialysis patients are prone to infection and higher mortality following sepsis. The virulence factors in the gut metagenome have not been well studied in hemodialysis patients, which could be employed by microorganisms to successfully thrive and flourish in their hosts. In this study, we performed shotgun metagenomics sequencing on fecal DNA collected from 16 control subjects and 24 hemodialysis patients. Our analysis shows that a number of microbial species, metabolic pathways, antibiotic resistance, and virulence factors were significantly altered in hemodialysis patients compared with controls. In particular, erythromycin resistance methylase, pyridoxamine 5-phosphate oxidase, and streptothricin-acetyl-transferase were significantly increased in hemodialysis patients. The findings in our study laid a valuable foundation to further elucidate the causative role of virulence factors in predisposing HD patients to infection and to develop treatment strategies to reduce the genetic capacities of antibiotic resistance and virulence factors in HD patients.

## Introduction

The increasing global epidemic of chronic kidney disease (CKD) and resultant end-stage renal disease (ESRD) continues to be a serious challenge for many countries (Zazzeroni et al., 2017). Increased translocation of bacterial-derived endotoxin into the circulation across an impaired intestinal barrier may contribute to systemic inflammation. Infection is the second major cause of hospitalization in the CKD population (Saran et al., 2019). Hemodialysis (HD) is a therapeutic procedure and a life-sustaining treatment for patients with kidney failure, which uses the extracorporeal circulation of a patient’s blood to ameliorate uremic syndrome. Powe et al. reported 11.7% of HD patients developed at least one episode of septicemia during 7 years of follow-up (Powe et al., 1999).

Human gut is colonized by trillions of microorganisms, which outnumber the host cells. The gut microbiota is essential for resisting pathogens and maintaining normal immune and metabolic homeostasis (Ley et al., 2006). Gut dysbiosis is characterized by the imbalance of microbial composition (Lin et al., 2021a). Gut dysbiosis is linked to various human diseases, which makes dysbiosis a key concept to understand how human gut microbiota contributes to diseases. Quantitative and functional changes in gut microbiota and microbial metabolites are reported in patients with CKD and ESRD (Barreto et al., 2009; Vaziri et al., 2013; Wong et al., 2014; Tang et al., 2015; Hobby et al., 2019; Wang et al., 2020; Lin et al., 2021b; Rysz et al., 2021). Aberrant gut microbiota has been reported to alter the host co-metabolism and immune response (Gao et al., 2021). Commensal bacteria play an important role in maintaining intestinal epithelial barrier by suppressing intestinal inflammation. Changes in the gut permeability result in compromised gut barrier function, which increases translocation of microbes and microbial products such as lipopolysaccharide into the systemic circulation. Uremia disrupts intestinal barrier and increases intestinal permeability in CKD (Magnusson et al., 1991). HD patients with sepsis have higher incidence, morbidity, and mortality (Sarnak and Jaber, 2000).

Gut microorganisms exploit all sources of resistance genes to develop the capacities to resist antibiotics introduced in clinical practice. Dialysis patients have higher prevalence and colonization with multidrug resistance organisms (Su et al., 2018). There is a need for more information on the antibiotic resistance in order to achieve better therapeutic effects for HD patients. A wide array of virulence factors is employed by microorganisms to successfully thrive and flourish in their hosts, often leading to fatal infections (Leitão, 2020). Elucidating the status of virulence factors in HD patients could help improve our understanding of the cellular and molecular mechanisms of bacterial pathogenesis in HD patients and to assess the efficacy of different empiric antibiotics in order to lower the morbidity and mortality in HD patients.

In order to characterize the functional alterations by gut microbiota, especially the antibiotic resistance and virulence factors in HD patients, we performed shotgun metagenomics analysis on fecal samples collected from 24 HD patients and 16 controls. Our analysis shows that a number of microbial species, metabolic pathways, antibiotic resistance, and virulence factors were significantly altered in HD patients.

## Materials and methods

### Patients

Data for this study are derived from a previous study, but the samples were collected from HD patients before Patiromer was administered (Gao et al., 2021). Inclusion criteria of HD patients were (1) maintaining HD for more than 60 days; (2) age ≥ 21 and ≤ 70 years; and (3) serum K+ >5.0 mEQ/L noted in three consecutive observations during the previous 3 months. Exclusion criteria include (1) use of prebiotics or probiotics during the past 2 months; (2) consumption of probiotic yogurt during the past 2 weeks; (3) use of antibiotics within the past 2 months; (4) chronic infection including hepatitis B and hepatitis C; (5) chronic GI condition other than constipation; (6) liver disease; (7) chronic inflammatory diseases; (8) presence of residual kidney function (urine output ≥200 ml/day); (9) advanced heart failure; (10) noncompliance with treatment; (11) use of proton pump inhibitors and immunosuppressive medications during the previous 3 months; and (12) malignancy, pregnancy, and participation in another intervention study. Control subjects with normal kidney function were recruited from the outpatient clinics at the George Washington University. Clinical and laboratory data were extracted from electronic medical records and confirmed with patients. Stool samples were collected by the participants themselves with a stool specimen collection kit (Fisherbrand Commode Specimen Collection System, Fisher Scientific, Waltham, MA, USA). All samples were processed, aliquoted, and stored at a −80°C freezer until analysis.

### Shotgun metagenomics sequencing

Metagenomics sequencing was performed at the Alkek Center for Metagenomics and Microbiome Research at Baylor College of Medicine. Briefly, total DNA was extracted from fecal samples using a DNeasy PowerSoil Kit (Qiagen, Germantown, MD, USA) followed by library construction using a KAPA Hyper kit (Kapa Biosystems, Wilmington, MA, USA). The NovaSeq 6000 platform was used for the metagenomics sequencing (Illumina, San Diego, CA, USA) with 2×150 bp paired-end read protocol.

### Metagenomics sequencing data analysis

Raw sequenced reads were paired-filtered using Trimmomatic (Bolger et al., 2014) version 0.38. After quality control, sequencing reads from the host were removed by Bowtie 2. Sequencing reads were then taxonomically profiled with MetaPhlAn2 with its default database, which contains about 1 million unique clade-specific maker genes identified from 17,000 reference genomes (Segata et al., 2012). To efficiently and accurately profile the relative abundance of microbial pathways, the metagenomic sequencing data were processed by the HUMAnN2 pipeline with the MetaCyc metabolic pathway database (Karp, 2002). ShortBRED was used to profile virulence factors with default settings, which will record a hit to a marker if the resulting alignment has at least 95% identity, and is at least as long as the minimum of the marker length or 95% of the read length (Kaminski et al., 2015). The count was normalized in the units of reads per kilobase of reference sequence per million sample reads (RPKMs). The normalized count was used for subsequent statistical analysis. Virulence Factors Database version 0.9.3 and Antibiotic Resistance Factors Database version 2017 was used in this study to profile virulence factors and antibiotic resistance factors, respectively. The raw sequencing data have been deposited to EBI metagenomics (accession number: PRJEB52094). The accession IDs are ERR9492489, ERR9492490, ERR9492491, ERR9492492, ERR9492493, ERR9492494, ERR9492495, ERR9492496, ERR9492497, ERR9492498, ERR9492499, ERR9492500, ERR9492501, ERR9492502, ERR9492503, ERR9492504, ERR9492505, ERR9492506, ERR9492507, ERR9492508, ERR9492509, ERR9492510, ERR9492511, ERR9492512, ERR9492513, ERR9492514, ERR9492515, ERR9492516, ERR9492517, ERR9492518, ERR9492519, ERR9492520, ERR9492521, ERR9492522, ERR9492523, ERR9492524, ERR9492525, ERR9492526, ERR9492527, and ERR9492528.

### Statistical analysis

Shannon index and Simpson index were calculated to evaluate alpha-diversity using the Microbiome package version 1.13.12 in R version 4.1.0. LEfSe was used to detect the significant microbial species, pathways, antibiotic resistance, and virulence factors (Segata et al., 2011). Spearman correlation analysis was performed between clinical parameters and microbial species, pathways, and virulence factors. Random forest model was built using the H2O platform (https://www.h2o.ai) to predict HD patients using microbial species, metabolic pathways, and virulence factors. The datasets were stratified split into training and test datasets (80:20) and we tuned the model by performing stratified fivefold cross-validation on the training set. Circos plot was generated *via* Circlize package in R version 4.1.0. Raw *p*-value less than 0.05 was considered as significant in this study. FDR was calculated using the fdr.adjust function in R.

## Results

### Patient characteristics

A total of 40 subjects were recruited in our study, namely, 24 HD patients and 16 control subjects. Patients’ characteristics are summarized in **Table 1**. The HD patient group consists of 12 (50%) male patients and 12 (50%) female patients with a mean age of 57 years, and a mean BMI of 32 kg/m^2^ (**Table 1**).

**Table 1:** Subject characteristics.

Patient variable	Controls	HD patients	*p*-value
Sex female	10 (67%)	12 (50%)	0.491
Age	64 ± 10	57 ± 10	0.041
Race			0.143
Asian	2 (13%)	0 (0%)	
Black	9 (60%)	17 (70%)	
Caucasian	4 (27%)	4 (17%)	
Other	0 (0%)	3 (13%)	
Body mass index (kg/m^2^)	30.7 ± 7.6	31.8 ± 6.3	0.642
Diabetes mellitus	6 (40%)	9 (38%)	0.999
Glucose (mg/dl)	143.0 ± 88.7	123.0 ± 58.6	0.455
Blood urea nitrogen (mg/dl)	12.5 ± 5.4	64.5 ± 14.5	<0.001
Serum creatinine (mg/dl)	0.9 ± 0.2	10.6 ± 1.9	<0.001
Sodium (mmol/L)	142.0 ± 2.9	139.0 ± 3.0	0.005
Potassium (mmol/L)	4.4 ± 0.4	5.7 ± 0.8	<0.001
Chloride (mmol/L)	103.0 ± 2.9	95.3 ± 3.3	<0.001
Carbon dioxide (mmol/L)	24.4 ± 2.7	20.2 ± 2.5	<0.001
Calcium (mg/dl)	9.5 ± 0.4	8.9 ± 0.6	0.001
EGFR	80.9 ± 19.0	5.4 ± 1.4	<0.001

Values of the control subjects were within the recommended normal range.

### Gut microbiota composition

**Figure 1:**
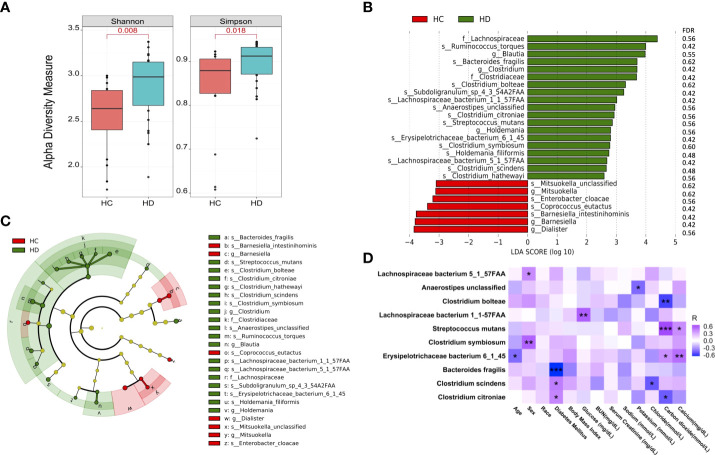
Microbial species are altered in HD patients. **(A)** Alpha-diversity of microbial community assessed by Shannon and Simpson index. **(B)** Significantly altered microbial species between HD patients and control subjects. **(C)** Cladogram of significantly altered microbes. **(D)** Correlations between the gut microbes with clinical data. *p-value < 0.05; **p-value < 0.01; ***p-value < 0.001. BUN: blood urea nitrogen.

The number of sequencing reads is shown in **Table S1**. In total, 341 microbial species were detected in this study (**Table S2**). Shannon and Simpson indexes were significantly increased in HD patients (**Figure 1A**). However, beta-diversity was not significantly altered as assessed by unweighted and weighted UniFrac distances (**Figure S1**). Significantly altered gut microbes between control subjects and HD patients are shown in the bar graph and cladogram (**Figures 1B** **, C**, **Table S3**). We examined the association between these significant microbes and clinical data in HD patients, among which 10 showed correlations with different clinical parameters (**Figure 1D**).

### Microbial pathways

**Figure 2:**
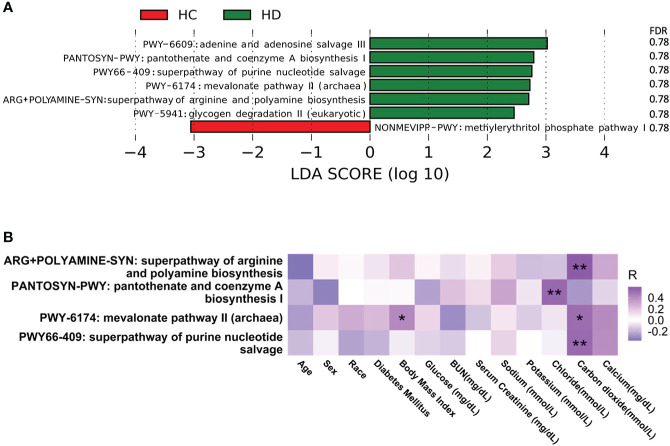
Alterations in microbial pathways. **(A)** Significantly altered microbial pathways between HD patients and control subjects. **(B)** Correlation between microbial pathways and clinical parameters. *p-value < 0.05; **p-value < 0.01. BUN, blood urea nitrogen.

Seven metabolic pathways were significantly altered between HD patients and control subjects, among which NONMEVIPP-PWY: methylerythritol phosphate pathway I decreased in HD patients, while the remaining pathways increased (**Figure 2A**, **Table S4**). Among these seven metabolic pathways, four pathways showed significant correlation with different clinical parameters (**Figure 2B**). The relative abundance of ARG+POLYAMINE-SYN: superpathway of arginine and polyamine biosynthesis, PWY-6174: mevalonate pathway II (archaea), and PWY66-409: superpathway of purine nucleotide salvage was positively associated with the level of carbon dioxide. The relative abundance of PANTOSYN-PWY: pantothenate and coenzyme A biosynthesis I was positively associated with the level of chloride. The relative abundance of PWY-6174: mevalonate pathway II (archaea) was positively associated with body mass index (BMI).

### Antibiotic resistance and virulence factors

**Figure 3:**
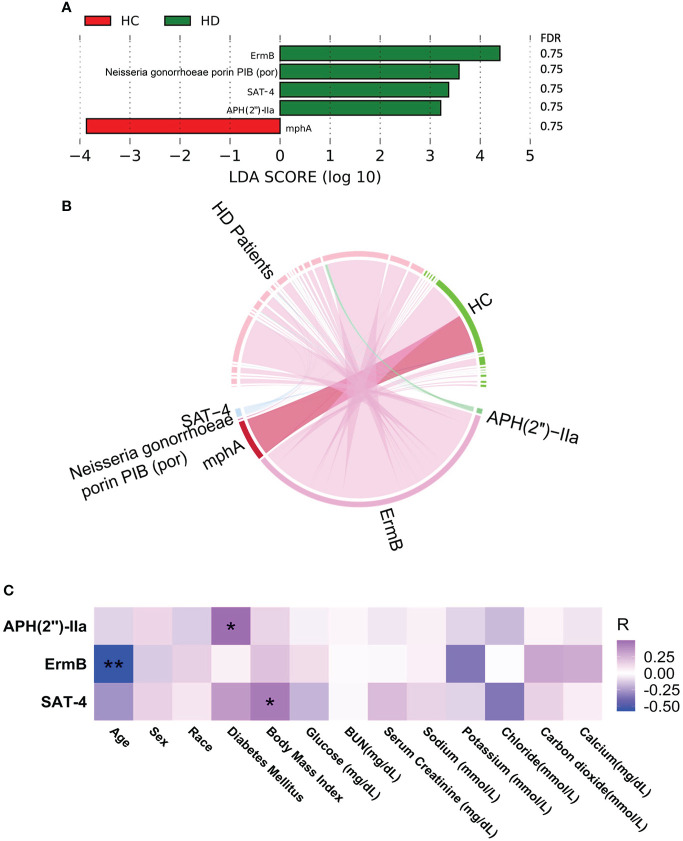
Alterations in virulence factors. **(A)** Significantly altered virulence factors between HD patients and control subjects. **(B)** Distribution of virulence factors in each sample. Data were visualized *via* Circlize package in R. **(C)** Correlations between virulence factors and clinical data. *p-value < 0.05; **p-value < 0.01. BUN, blood urea nitrogen.

**Figure 4:**
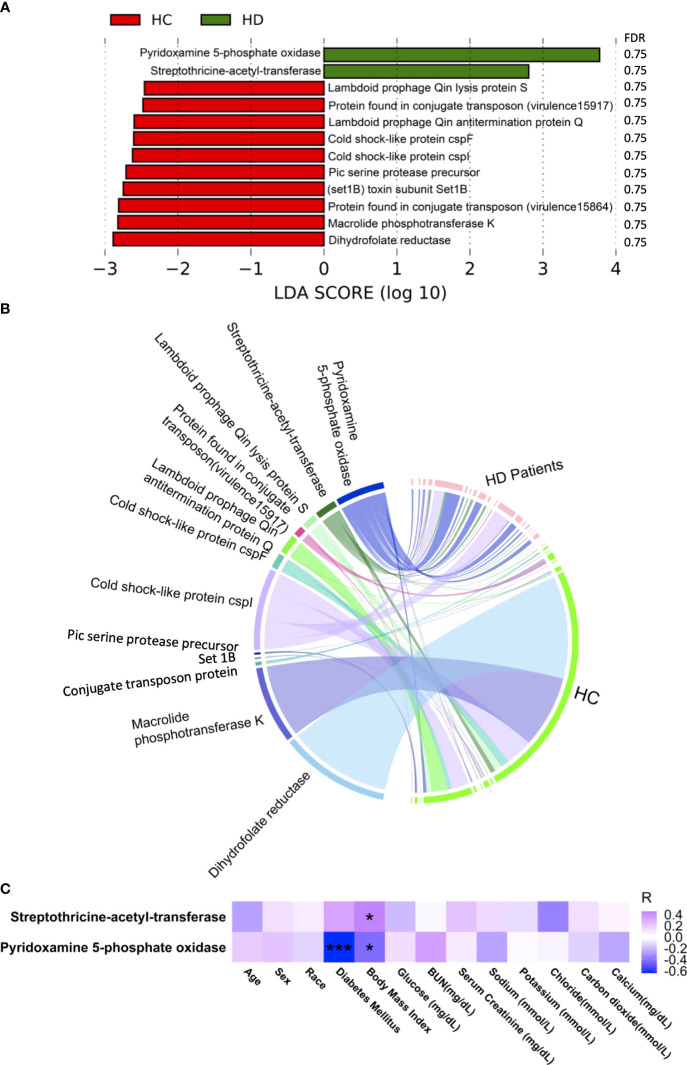
Alterations in antibiotic resistance factors. **(A)** Significantly altered antibiotic resistance factors between HD patients and control subjects. **(B)** Distribution of antibiotic resistance factors in each sample. Data were visualized *via* Circlize package in R. **(C)** Correlations between antibiotic resistance factors and clinical data. *p-value < 0.05; ***p-value < 0.001. BUN, blood urea nitrogen.

Five antibiotic resistance factors were significantly altered in HD patients compared with controls, among which the level of four were higher in HD patients, namely, *ErmB*, *Neisseria gonorrhoeae porin PIB (por)*, *SAT-4*, and *APH(2’’)-IIa* (**Figures 3A** and **Figure S2**, **Table S5**). Among these significant antibiotic resistance factors, erythromycin resistance methylase (erm) was the most abundant (**Figure 3B**). Three antibiotic resistance factors were significantly correlated with various clinical parameters (**Figure 3C**). The possible resistance mechanism for these antibiotic resistance genes is shown in **Table S6**. A total of 12 virulence factors were significantly altered, among which the relative abundance of pyridoxamine 5-phosphate oxidase and streptothricin-acetyl-transferase was higher in HD patients (**Figures 4A** and **Figure S3**, **Table S7**). The microbial origin of these virulence factors is shown in **Table S8**. Among these significant virulence factors, dihydrofolate reductase was the most abundant (**Figure 4B**). Streptothricin-acetyl-transferase was positively associated with BMI, while pyridoxamine 5-phosphate oxidase was negatively correlated with BMI and diabetes mellitus (**Figure 4C**).

### Random forest model distinguishes HD patients from controls

**Figure 5:**
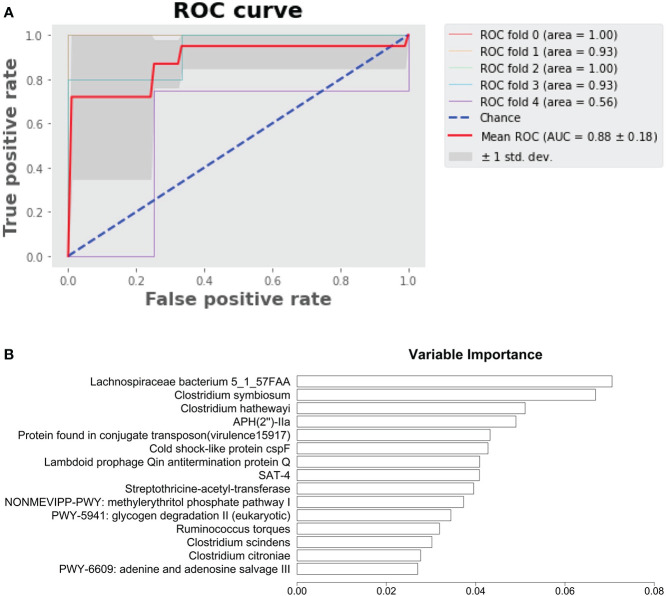
Prediction of HD patients with random forest model. **(A)** Random forest model predicting HD patients from control subjects. **(B)** Variable importance.

We developed a random forest model to predict HD patients from control subjects using microbial species, pathways, antibiotic resistance, and virulence factors. The area under the curve achieved 0.88 (**Figure 5A**). The variable importance of top 10 variables is shown in **Figure 5B**, with Lachnospiraceae bacterium 5_1_57FAA emerging as the most important variable.

## Discussion

Gut microbiota in HD patients was altered in community composition and function compared to individuals without kidney disease in our study. A previous study showed that dysbiosis of the gut microbiota in patients with CKD is characterized by an increase in the types of bacteria with proteolytic fermentation activity, such as *Bacteroides* and *Clostridium*, and circulating uremic toxins produced by the fermentation of nitrogen-containing compounds (Rukavina Mikusic et al., 2020). Consistently, *B. fragilis* was increased in HD patients compared with control subjects in our study. An increase of *Bacteroidetes* has been reported previously in HD patients compared with healthy individuals (Crespo-Salgado et al., 2016). *B. fragilis* is a part of the normal microbiota in human colon. *Bacteroides* spp. involved in the maintenance of gut mucosal barrier function were enriched in patients with both type 1 and type 2 diabetes mellitus (Kanbay et al., 2018). Polysaccharide A produced by *B. fragilis* activates T cell-dependent immune responses (Mazmanian et al., 2005). *B. fragilis* has also been shown to cause clinically significant infection (Elsaghir and Reddivari, 2021). An unusual case of clavicular osteomyelitis caused by *B. fragilis* was reported in a patient undergoing maintenance HD following colonoscopy (Kambhampati et al., 2011).

Several species in *Clostridium* genus were increased in HD patients in our study. Increases in various opportunistic pathogens, including *Clostridium hathewayi*, have been reported in patients with type 2 diabetes mellitus (Hyland and Stanton, 2016). *Ruminococcus torques* was also increased in HD patients in our study. *Ruminococcus* spp. increase in various clinical situations (Sato et al., 2021). *R. torques* is known to degrade gastrointestinal mucin and plays a role in the pathophysiology of Crohn’s disease (Joossens et al., 2011). *R. torques* also increases in children with autism spectrum disorder (Wang et al., 2013).

Dysregulation of gut microbiota leads to the alterations of microbial pathways in our study, which were mainly associated with nucleotide biosynthesis. Purine nucleotide salvage is a secondary pathway of purine nucleotide anabolism. This metabolic pathway has important physiological significance for some tissues that lack the main pathway, such as leukocytes and platelets, brain, bone marrow, and spleen. A previous study has also shown that pathogens require nucleotide biosynthesis to establish a successful infection (Samant et al., 2008).

In addition to the metabolic pathways, we specifically examined the genes encoding antibiotics resistance and the virulence factors in the gut metagenome. Increase of genes encoding erythromycin resistant methylase was found in HD patients, compared with control subjects in our study. Erythromycin-resistant methylase is encoded by a series of structurally related *erm* genes in different bacterial species, which methylates ribosomal RNA, resulting in high-level resistance to macrolide (Pechère, 2001). Six-week eradication therapy significantly increased the richness of *erm (B)* gene in the gut microbiota (Hsu et al., 2019). Meanwhile, several cold shock-like proteins including cspF were decreased in HD patients in the present study. CspF is a member of the Csp family, which counteracts cold shock effects by serving as nucleic acid chaperones that may prevent the formation of secondary structures in mRNA at low temperature and thus facilitate the initiation of translation (Keto-Timonen et al., 2016).

CKD can range from the beginning stage to advanced stages. However, this study only included HD patients and control subjects without kidney disease. Another limitation of this study is the small sample size. Therefore, the findings from this study need to be validated in a larger and independent patient cohort with different stages of CKD patients. The findings in our study laid a valuable foundation to further elucidate the causative role of virulence factors in HD patients and to develop treatment strategies to reduce the genetic capacity of virulence factors in HD patients.

## Data availability statement

The data presented in the study are deposited in the EBI metagenomics repository (https://www.ebi.ac.uk/ena), accession number PRJEB52094.

## Ethics statement

The studies involving human participants were reviewed and approved by George Washington University institutional review board. The patients/participants provided their written informed consent to participate in this study.

## Author contributions

XS analyzed the metagenomics data; XS, BG wrote the manuscript; AS, AI, YA, WS revised the manuscript; DR analyzed the clinical data.

## Funding

This study is supported in part by Relypsa Inc, Redwood, California and from NIDDK grant U01DK099914 and R01DK125256.

## Acknowledgments

We acknowledge the High Performance Computing Center of Nanjing University of Information Science & Technology for their support of this work.

## Conflict of interest

The authors declare that this study received funding from Relypsa Inc. The funder was not involved in the study design, collection, analysis, interpretation of data, the writing of this article or the decision to submit it for publication.

## Publisher’s note

All claims expressed in this article are solely those of the authors and do not necessarily represent those of their affiliated organizations, or those of the publisher, the editors and the reviewers. Any product that may be evaluated in this article, or claim that may be made by its manufacturer, is not guaranteed or endorsed by the publisher.

## Supplementary material

Supplementary Figure 1 Beta-diversity was not altered in different groups. **(A)** Unweighted UniFrac distance. **(B)** Weighted UniFrac distance. HC, healthy controls; HD, HD patients.
