# Supplementary figures and images for: Alterations of gut microbial pathways and virulence factors in hemodialysis patients
Source: Front Cell Infect Microbiol. 2022 Aug 26;12:904284. doi: 10.3389/fcimb.2022.904284 (PMC9461950; doi:10.3389/fcimb.2022.904284)

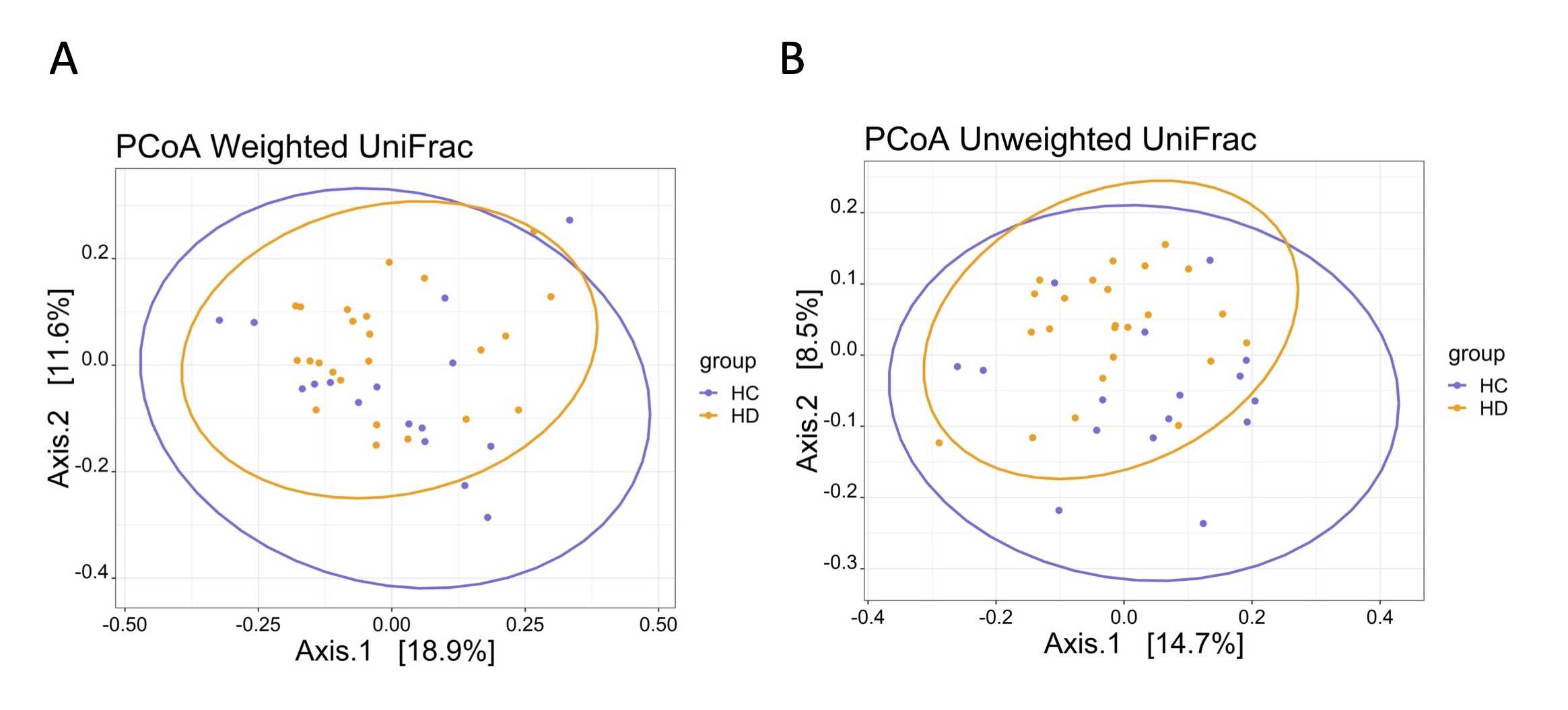

Supplement: Supplementary Figure 1 — Beta-diversity was not altered in different groups. (A) Unweighted UniFrac distance. (B) Weighted UniFrac distance. HC, healthy controls; HD, HD patients. [file Image_1.jpeg]
